# Comparison of lung ultrasound scores with clinical models for predicting bronchopulmonary dysplasia

**DOI:** 10.1007/s00431-023-04847-y

**Published:** 2023-02-09

**Authors:** Zhenyu Li, Xin Mu, Dan Dang, Xiaoming Lv, Shuyu Si, Yiyi Guo, Hui Wu

**Affiliations:** Department of Neonatology, The First Hospital of Jilin University, No. 1 Xinmin Street, Changchun, Jilin China

**Keywords:** Bronchopulmonary dysplasia (BPD), Lung ultrasound score (LUS), Gestational age (GA), Prediction

## Abstract

Lung ultrasound scores (LUSs) have been demonstrated to accurately predict moderate-to-severe bronchopulmonary dysplasia (msBPD). This study attempted to explore the additional value of LUSs for predicting msBPD compared to clinical multivariate models in different gestational age (GA) groups. The study prospectively recruited preterm infants with GA < 32 weeks. Lung ultrasound was performed on days 3, 7, 14, and 21 after birth. A linear mixed-effects regression model was used to evaluate LUS evolution in infants born before and after 28 weeks. The receiver operating characteristic (ROC) procedure was used to analyze the reliability of LUS and clinical multivariable models for predicting msBPD. The optimal time to predict msBPD in all infants was 7 days with a cut-off point of 5 (area under the ROC (AUROC) curve: 0.78, 95% confidence interval (CI): 0.71–0.84). In infants with GA ≥ 28 weeks, LUSs provided a moderate diagnostic accuracy for all four time points (AUROC curve: 0.74–0.78), and the AUROC curve for the clinical multivariable model on day 14 was 0.91 (95% CI: 0.84–0.96), which was significantly higher than that of LUSs (AUROC curve: 0.77, 95% CI: 0.68–0.85, *P* < 0.05). In infants born at 23–27 weeks, LUSs showed a low diagnostic accuracy with higher cut-off points to predict msBPD, and the AUROC curve for GA to predict msBPD was 0.75 (95% CI: 0.59–0.85), providing diagnostic accuracy similar to that of LUSs.

*  Conclusion*: The contribution of LUSs to predict msBPD in infants with different GAs remains controversial and requires further investigation.

**Table:** 

**What is Known:** *• Lung ultrasound scores (LUSs) have been demonstrated to accurately predict moderate-to-severe bronchopulmonary dysplasia in infants with gestational age (GA)＜32 weeks.*	
**What is New:** *• The LUSs evolution differed between extremely preterm infants born before 28 weeks and preterm infants born at 28–32 weeks of gestation.* *• LUSs provided similar moderate predictive performance as GA-adjusted LUS and clinical multivariate models in infants born after 28 weeks, while LUSs seem to be less helpful in infants born before 28 weeks.*

**Supplementary Information:**

The online version contains supplementary material available at 10.1007/s00431-023-04847-y.

## Introduction

Bronchopulmonary dysplasia (BPD) is one of the most common and severe sequelae in very preterm infants, and several studies have developed clinical prediction models to guide patient management [1, 2]. A web-based prediction model from the National Institutes of Health (NIH) reported areas under the receiver operating characteristic (ROC) (AUROC) curve of 0.81, 0.82, and 0.83 on days 3, 7, and 14 of postnatal age, respectively, for BPD prediction in infants born at 23–30 weeks [3]. In recent years, the application of lung ultrasonography in neonatal intensive care units (NICUs) has considerably increased due to its rapid acquisition, portability, lack of radiation exposure, excellent diagnostic accuracy, and reproducibility in following the disease progress [4–9]. Previous studies have revealed that lung ultrasound scores (LUSs) in the first 14 days after birth are useful for predicting BPD, with an AUROC curve of 0.7–0.9 in preterm infants [6, 9, 10]. Extremely preterm infants born before 28 weeks of gestation benefit the most from accurate early prediction of BPD; however, recent studies have expressed concerns about the accuracy of LUSs for predicting BPD in this population. The evolution of LUSs in this population is significantly different from that in infants with gestational ages (GAs) of 28–30 weeks and 31–33 weeks, and a recent study found that the predictive value of LUSs may be insignificant in the multivariate models in which GA was the main predictor in this population [11, 12].

Therefore, this study aimed to evaluate the predictive value of LUS for the development of moderate-to-severe BPD (msBPD) at different time points in infants born before and after 28 weeks. Additionally, we constructed multivariate regression models using available clinical variables to assess the utility of LUSs.

## Materials and methods

This prospective, observational, longitudinal diagnostic accuracy cohort study was conducted between January 2020 and July 2021 at Jilin University First Hospital, which has extensive experience in the use of neonatal ultrasound in China. This study was approved by the hospital’s institutional review board (clinical trial registered with www.chictr.org.cn [chiCTR1900023869]), and parental consent was obtained upon NICU admission. All procedures were performed in accordance with the Declaration of Helsinki.

This study enrolled preterm infants with GA < 32 weeks who were admitted to the NICU on the day of birth. The exclusion criteria were the following: (1) complex congenital malformations or chromosomal abnormalities, (2) congenital lung diseases or congenital heart defects, and (3) death before 36 weeks postmenstrual age.

All enrolled infants underwent lung ultrasonography performed by two ultrasound physicians at day 3 (D3), day 7 (D7, ± 1 day), day 14 (D14, ± 1 day), and day 21 (D21, ± 2 days) of postnatal age. The procedure was performed after at least 30 min in the supine position, following a standardized protocol [13]. All ultrasonographic images and videos were digitally recorded, anonymized, and reviewed by a senior independent ultrasonographer blinded to patients’ clinical information. Lung ultrasonography was performed with a linear probe (9–12 MHz) according to availability. The LUS calculation was based on the description of Brat et al. [6, 9], and the total score ranged from 0 to 18.

Respiratory data (PtcO2 and type of respiratory support) were collected on the same day in which the LUS was calculated. The ventilatory policy is shown in the supplementary material. PtcO2 was estimated with adequately calibrated transcutaneous oxygen tension monitoring devices (TCM4, Radiometer, Denmark). Respiratory support was defined as (i) invasive mechanical ventilation, (ii) non-invasive ventilation, (iii) oxygen (high-flow or low-flow oxygen), or (iv) none. If more than one respiratory support was recorded during the day, the highest form of ventilation was used. Additionally, given that the duration of hemodynamically significant patent ductus arteriosus (hsPDA) is associated with an increased risk of BPD [14, 15], we dynamically recorded the presence of hsPDA using echocardiography within 1 day of lung ultrasonography: left atrial-aortic root ratio > 1.3, PDA size > 1.5 mm, and a ductal left-to-right shunt [16].

The study’s primary outcome was msBPD, defined as the requirement for oxygen or respiratory support at 36 weeks postmenstrual age or at the time of discharge, and the secondary outcome was any grade of BPD defined by the NIH in 2001 [17].

### Statistical analysis

The sample size was calculated using PASS software (NCSS, LLC, Kaysville, UT, USA). Approximately 35% of preterm infants with GA < 32 weeks developed msBPD in our NICU during the first 6 months of the study. An AUROC curve of ≥ 0.7, as previously published, and an *α* error of 0.05 and 90% power were targeted [18]. Based on this calculation, the required sample size was 94 infants.

Normally distributed continuous variables were expressed as the mean ± standard deviation (SD) and compared with Student’s *t* test, while non-normally distributed continuous variables were summarized as medians and interquartile values and compared with Mann–Whitney test. Categorical variables were summarized as counts and percentages and compared with chi-square (*χ*^2^) or Fisher’s test, as appropriate. LUSs were compared between groups using repeated-measures analysis of variance. The ROC procedure was used to analyze the reliability of LUSs for predicting msBPD at different time points. The AUROCs were compared using DeLong’s test, and the optimal cut-off points were determined using Youden’s method.

Data analysis was based on different GA groups. First, the predictive performance of LUSs for msBPD was evaluated according to different GA groups. Second, the enrolled infants were allocated to one of two GA groups (23–27 weeks and 28–32 weeks) to evaluate the LUS evolution and the contribution of different predictors of msBPD. Linear multilevel mixed-effects regression was used to estimate the predictive model of LUS evolution for msBPD as an effect-modifying and interaction variable. To evaluate the utility of LUSs as predictive models, multivariate logistic regression was applied for msBPD prediction using available clinical covariates without multicollinearity and compared with LUSs and GA-adjusted LUSs. Model 1 included GA alone, model 2 included LUS alone, model 3 included GA and LUS (GA-adjusted LUS), and model 4 included GA, sex, and presence of hsPDA, type of respiratory support, and PtcO2 on the day of lung ultrasonography. Multicollinearity between clinical covariates was examined according to tolerance and variance inflation factor. Multicollinearity between clinical covariates was examined according to tolerance and variance inflation factor. GA was chosen as a covariate because it plays a prominent role in the development of BPD [19], and birth weight was excluded because it creates relevant multicollinearity with GA. The predictive performances of the selected models were assessed using the AUROC curve and 95% confidence interval (CI), and goodness-of-fit was evaluated using the Hosmer–Lemeshow test.

Analyses were performed using Stata version 16.0 (Stata Corp., College Station, TX, USA), MedCalc version 19.0 (MedCalc bvba, Ostend, Belgium), and GraphPad Prism V8.0 (San Diego, CA, USA).

## Results

**Fig. 1:**
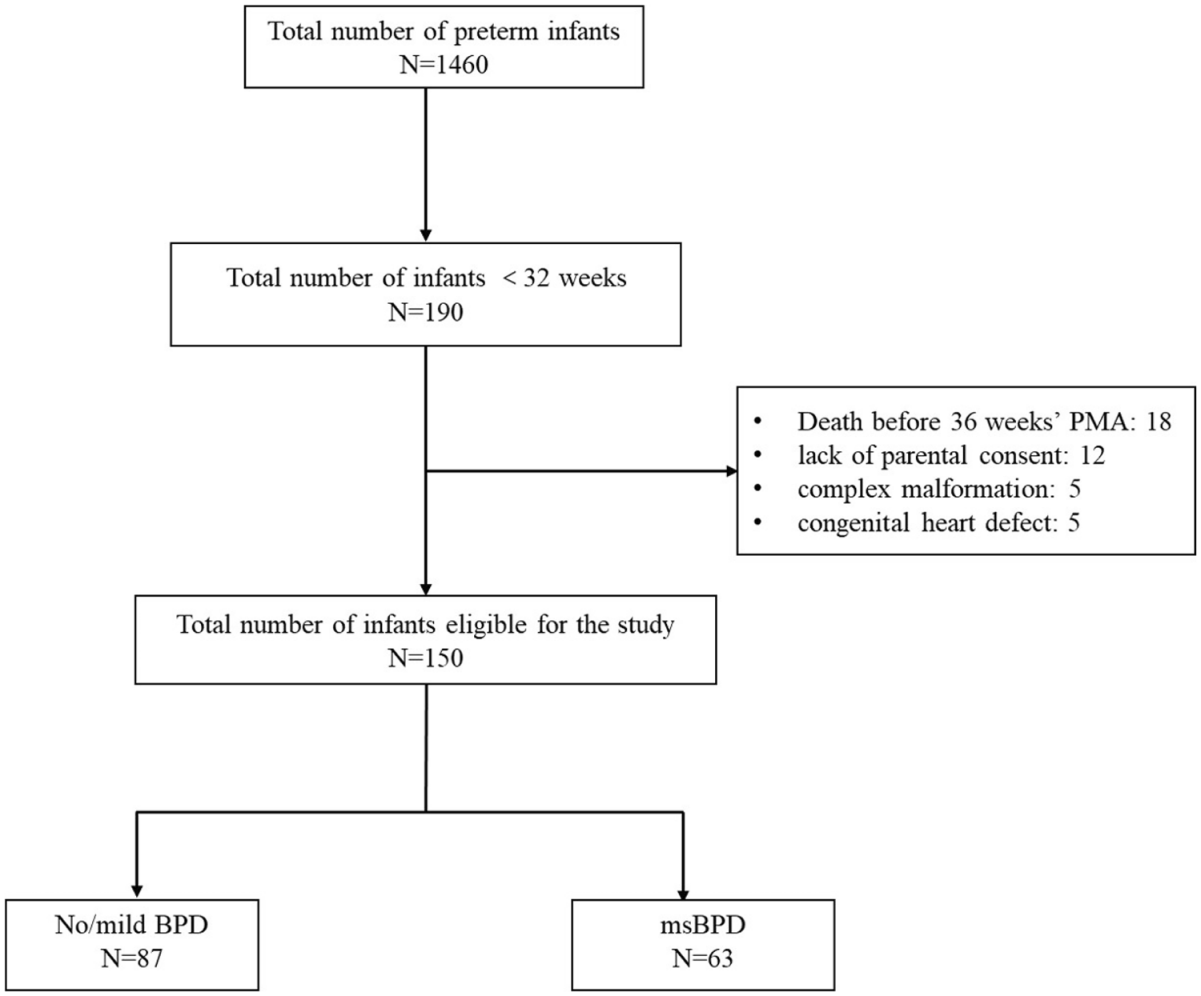
Study chart

There were 190 eligible infants during the study period (Fig. 1). The clinical characteristics of the enrolled infants (*n* = 150) are described in Table 1; 63 (42%) were diagnosed with msBPD, and 87 (58%) had no/mild BPD. The mean GA and birth weight of enrolled infants were 28.70 (SD: 1.59) weeks and 1176.5 (SD: 224.6) g, respectively. Infants who developed msBPD were more preterm, more likely to have hsPDA, had higher CRIB-II scores, and required longer respiratory support. There were 43 (28.7%) and 107 (71.3%) infants born at 23–27 weeks and ≥ 28 weeks of gestation, respectively, and their clinical characteristics are described in Supplemental Table 1.

**Table 1:** Basic population details

Characteristic	Total (*n* = 150)	Non-mild BPD (*n* = 87)	msBPD (*n* = 63)	*P* value
GA, weeks, mean (SD)	28.70 (1.59)	29.4 (1.12)	27.7 (1.63)	< 0.001
BW, g, mean (SD)	1176.5 (224.6)	1276.3 (171.8)	1038.6 (216.7)	< 0.001
Male, no. (%)	77 (51.3)	45 (51.7)	32 (50.8)	0.910
SGA neonates, no. (%)	5 (3.3)	2 (2.3)	3 (4.8)	0.650
Cesarean delivery, no. (%)	57 (38)	35 (40.2)	22 (34.9)	0.508
CRIB-II score, median (IQR)	8 (6–10)	6 (4–8)	10 (8–12)	< 0.001
Antenatal steroids, no. (%)	97 (64.7)	57 (65.5)	40 (63.5)	0.798
PPROM, no. (%)	53 (35.3)	37 (42.5)	16 (25.4)	0.030
Clinical chorioamnionitis, no. (%)	34 (22.7)	19 (21.8)	15 (23.8)	0.776
MV for over 24 h, no. (%)	107 (71.3)	46 (52.9)	61 (96.8)	< 0.001
hsPDA, no. (%)	90 (60)	36 (41.4)	54 (85.7)	< 0.001

*P* values are calculated with chi-square (*χ*^2^) or Fisher’s test and Student’s *t* test or Mann–Whitney test, as appropriate

*BW* birth weight, *GA* gestational age, *hsPDA* hemodynamically significant patent ductus arteriosus, *IQR* interquartile range, *MV* mechanical ventilation, *PPROM* preterm premature rupture of membranes, *SD* standard deviation, *SGA* small for gestational age

### Diagnostic accuracy of the LUSs in all infants

**Fig. 2:**
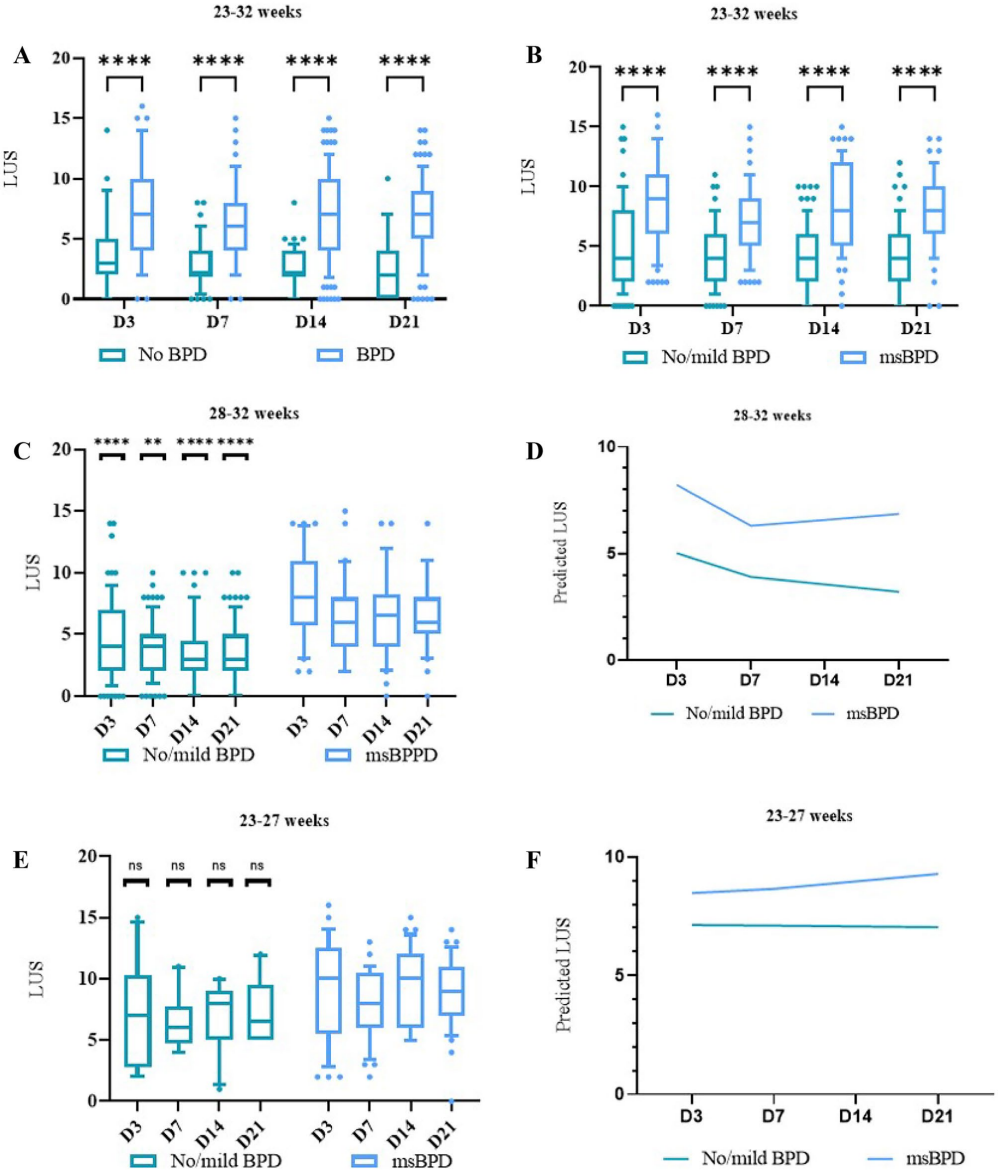
Lung ultrasound scores (LUSs) at different time points. Statistical significance: *P < 0.05, **P < 0.01, ***P < 0.001, ****P < 0.0001. **A** Boxplot showing LUSs at D3, at D7, at D14, and at D21 in infants with BPD and without BPD. **B** Boxplot showing LUSs at D3, at D7, at D14, and at D21 in infants with msBPD and no/mild BPD. **C**, **D** Boxplot and predicted LUS evolution over time in infants born at 23–27 weeks of gestation. **E**, **F** Boxplot and predicted LUS evolution over time in infants born at 28–32 weeks of gestation

This cohort had significant differences in LUSs between infants with and without BPD and between infants with msBPD and with no/mild BPD from D3 to D21 (Fig. 2A, B; *P* < 0.0001, between-participant comparisons). LUSs had good diagnostic accuracy for predicting any BPD and msBPD on D3, D7, D14, and D21, as shown in Table 2 and Supplemental Table 2. The optimal time for BPD prediction was D14 with a cut-off point of 4 (sensitivity: 72.9%, specificity: 90.7%, AUROC curve: 0.87, 95% CI: 0.81–0.92). In comparison, the optimal times for msBPD prediction were D7 and D14, with the cut-off points of 5 and 4 for each time (sensitivity: 71.4% and 85.7%, specificity: 73.6% and 67.8%; AUROC curve: 0.78 [95% CI: 0.71–0.84] and 0.82 [95% CI: 0.75–0.88], respectively).

**Table 2:** The predictive ability of LUS for moderate-to-severe bronchopulmonary dysplasia (msBPD) in different GA groups

	Cut-off point	AUROC curve (95% CI)	Sensitivity, % (95% CI)	Specificity, % (95% CI)	+LR (95% CI)	−LR (95% CI)	PPV, % (95% CI)	NPV, % (95% CI)
**23–32 weeks**								
D3	> 5	0.76 (0.69–0.83)	76.2 (63.8–86.0)	62.1 (51.0–72.3)	2.01 (1.5–2.7)	0.38 (0.2–0.6)	59.3 (51.8–66.3)	78.3 (69.2–85.2)
D7	> 5	0.78 (0.71–0.84)	71.4 (58.7–82.1)	73.6 (63.0–82.4)	2.70 (1.8–4.0)	0.39 (0.3–0.6)	66.2 (57.1–74.2)	78.0 (70.2–84.3)
D14	> 4	0.82 (0.75–0.88)	85.7 (74.6–93.3)	67.8 (56.9–77.4)	2.66 (1.9–3.7)	0.21 (0.1–0.4)	65.9 (58.3–72.7)	86.8 (77.9–92.4)
D21	> 5	0.81 (0.74–0.87)	76.2 (63.8–86.0)	72.4 (61.8–81.5)	2.76 (1.9–4.0)	0.33 (0.2–0.5)	66.7 (58.1–74.3)	80.8 (72.6–86.9)
**28–32 weeks**								
D3	> 5	0.77 (0.67–0.84)	76.7 (57.7–90.1)	64.9 (53.2–75.5)	2.19 (1.5–3.1)	0.36 (0.2–0.7)	46.0 (37.2–55.0)	87.7 (78.5–93.3)
D7	> 5	0.74 (0.65–0.82)	60.0 (40.6–77.3)	77.9 (67.0–86.6)	2.72 (1.6–4.5)	0.51 (0.3–0.8)	51.4 (38.8–63.8)	83.3 (76.0–88.7)
D14	> 4	0.77 (0.68–0.85)	70.0 (50.6–85.3)	75.3 (64.2–84.4)	2.84 (1.8–4.5)	0.4 (0.2–0.7)	52.5 (41.2–63.5)	86.6 (78.6–91.9)
D21	> 4	0.78 (0.68–0.85)	80.0 (61.4–92.3)	68.8 (57.3–78.9)	2.57 (1.8–3.7)	0.29 (0.1–0.6)	50.0 (40.7–59.3)	89.8 (81.0–94.8)
**23–27 weeks**								
D3	> 9	0.66 (0.51–0.79)	57.6 (39.2–74.5)	70.0 (34.8–93.3)	1.92 (0.7–5.2)	0.61 (0.3–1.1)	86.4 (70.2–94.5)	33.3 (22.1–46.9)
D7	> 7	0.69 (0.54–0.83)	66.7 (48.2–82.0)	80.0 (44.4–97.5)	3.33 (0.9–11.8)	0.42 (0.2–0.7)	91.7 (75.7–97.5)	42.1 (29.1–56.3)
D14	> 9	0.70 (0.54–0.83)	54.5 (36.4–71.9)	90.0 (55.5–99.7)	5.45 (0.8–35.9)	0.51 (0.3–0.8)	94.7 (73.2–99.2)	37.5 (28.1–47.9)
D21	> 7	0.70 (0.54–0.83)	66.7 (48.2–82.0)	70.0 (34.8–93.3)	2.22 (0.8–5.9)	0.48 (0.3–0.9)	88.0 (73.4–95.1)	38.9 (25.3–54.4)

*AUROC* area under the receiver operating characteristic, *CI* confidence interval, *LR* likelihood ratio, *PPV* positive predictive values, *NPV* negative predictive values

### Diagnostic accuracies of LUSs and LUS evolution in infants born at 28–32 weeks

Among infants with GA ≥ 28 weeks, LUS showed a similar moderate predictive accuracy for msBPD on D3 (AUROC curve: 0.77, 95% CI: 0.67–0.84), D7 (AUROC curve: 0.74, 95% CI: 0.65–0.82), D14 (AUROC curve: 0.77, 95% CI: 0.68–0.85), and D21 (AUROC curve: 0.78, 95% CI: 0.68–0.85) (Table 2). Infants who developed msBPD had significantly higher LUSs than those with no/mild BPD from D3 to D21 (Fig. 2C). The LUS evolution predicted is displayed in Fig. 2D, showing that LUSs decreased rapidly during the first week of life, followed by a slow decrease, in infants with no/mild BPD. In contrast, LUSs remained high and stable in infants with developing msBPD. The statistics of the linear multilevel mixed-effects regression models are shown in Supplemental Table 2.

There was no multicollinearity between independent variables in model 4 (tolerance > 0.1 and variance inflation factor < 10 for GA, type of respiratory support, and PtcO2) (Supplemental Table 3). PtcO2 values were allocated to one of three groups: (i) hypoxemia (PtcO2 < 50 mmHg), (ii) normoxemia (PtcO2 50–80 mmHg), or (iii) hyperoxemia (PtcO2 > 80 mmHg). In the five-factor model, the variables most associated with an increased risk of msBPD changed depending on the postnatal day (Table 3). Low GA (odds ratio (OR): 0.43; 95% CI: 0.24–0.78; *P* < 0.01) and the presence of hsPDA (OR: 3.94; 95% CI: 1.42–10.9; *P* < 0.01) were associated with the development of msBPD on D3, while higher levels of respiratory support (OR: 0.14; 95% CI: 0.04–0.45; *P* < 0.01), lower PtcO2 values (OR: 0.24; 95% CI: 0.08–0.76; *P* < 0.01), and the presence of hsPDA (OR: 5.96; 95% CI: 1.55–22.9; *P* < 0.01) were associated with msBPD on D14. LUS provided a diagnostic accuracy similar to that of GA-adjusted LUS and the clinical five-factor model (model 4) on D3 (AUROC curve: 0.77 [95% CI: 0.67–0.84] vs 0.81 [95% CI: 0.72–0.88], *P* = 0.18; AUROC curve: 0.77 [95% CI: 0.67–0.84] vs 0.78 [95% CI: 0.69–0.86], *P* = 0.84) and D7 (AUROC curve: 0.74 [95% CI: 0.65–0.82] vs 0.77 [95% CI: 0.67–0.84], *P* = 0.42; AUROC curve: 0.74 [95% CI: 0.65–0.82] vs 0.82 [95% CI: 0.74–0.89], *P* = 0.19). The AUROC curve for the clinical five-factor model on D14 was 0.91 (95% CI: 0.84–0.96), which was significantly higher than that of LUSs (AUROC curve: 0.77, 95% CI: 0.68–0.85, *P* < 0.05) and GA-adjusted LUSs (AUROC curve: 0.80, 95% CI: 0.71–0.87, *P* < 0.05) on the same day (Table 4).

**Table 3:** Multivariate analysis to predict msBPD in infants born at 23–27 weeks and 28–32 weeks

Variable	D3		D7		D14	
	OR (95% CI)	*P* value	OR (95% CI)	*P* value	OR (95% CI)	*P* value
**28–32 weeks**						
GA	0.43 (0.24–0.78)	0.005	0.55 (0.29–1.05)	0.071	0.49 (0.24–1.03)	0.060
Sex	0.77 (0.29–2.03)	0.604	0.65 (0.23–1.90)	0.436	0.83 (0.26–2.67)	0.760
hsPDA	3.94 (1.42–10.9)	0.008	2.48 (0.84–7.33)	0.101	5.96 (1.55–22.9)	0.009
Respiratory support	2.15 (0.79–5.84)	0.131	9.36 (2.87–30.5)	< 0.001	6.94 (2.21–21.7)	0.015
PtcO2	0.62 (0.27–1.44)	0.270	1.03 (0.44–2.39)	0.949	0.24 (0.08–0.76)	0.019
**23–27 weeks**						
GA	0.19 (0.04–0.85)	0.029	0.14 (0.03–0.68)	0.014	0.25 (0.05–1.22)	0.087
Sex	0.48 (0.08–2.79)	0.418	0.68 (0.12–3.96)	0.670	0.27 (0.03–2.17)	0.219
hsPDA	2.87 (0.51–16.4)	0.234	5.74 (0.75–43.7)	0.091	13.4 (0.70–80.2)	0.084
Respiratory support	0.89 (0.09–8.47)	0.922	0.43 (0.05–3.51)	0.432	4.21 (0.36–48.9)	0.443
PtcO2	1.50 (0.38–5.85)	0.561	1.63 (0.31–8.60)	0.563	0.96 (0.19–4.94)	0.966

All models showed the Hosmer–Lemeshow test with *P* > 0.05

*GA* gestational age, *hsPDA* hemodynamically significant patent ductus arteriosus, *OR* odds ratio

**Table 4:** AUROC curves from different models to predict msBPD in infants born before 32 weeks of gestation

Models	AUROC curve (95% CI)		
	D3	D7	D14
28–32 weeks			
GA (model 1)	0.67 (0.58–0.76)	0.67 (0.58–0.76)	0.67 (0.58–0.76)
LUS (model 2)	0.77 (0.67–0.84)	0.74 (0.65–0.82)	0.77 (0.68–0.85)
GA-adjusted LUS (model 3)	0.81 (0.72–0.88)	0.77 (0.67–0.84)	0.80 (0.71–0.87)
Clinical multivariate model (model 4)	0.78 (0.69–0.86)	0.82 (0.74–0.89)	0.91 (0.84–0.96)
23–27 weeks			
GA (model 1)	0.75 (0.59–0.86)	0.75 (0.59–0.86)	0.75 (0.59–0.86)
LUS (model 2)	0.66 (0.51–0.79)	0.69 (0.54–0.83)	0.70 (0.54–0.83)
GA-adjusted LUS (model 3)	0.79 (0.64–0.90)	0.82 (0.67–0.92)	0.76 (0.61–0.88)
Clinical multivariate model (model 4)	0.83 (0.68–0.92)	0.80 (0.65–0.91)	0.84 (0.69–0.93)

In the upper table, *P* < 0.05 comparing AUROC curve from model 2 vs model 4 and model 3 vs model 4 on D14, and others *P* > 0.05; in the lower table, *P* > 0.05 comparing AUROC curve from model 1 vs model 2, model 1 vs model 3, model 1 vs model 4, model 2 vs model 3, model 2 vs model 4, and model 3 vs model 4 for all 3 time points

*GA* gestational age, *LUS* lung ultrasound score, *AUROC* area under the receiver operating characteristic, *CI* confidence interval

### Diagnostic accuracies of LUSs and LUS evolution in infants born at 23–27 weeks

In infants born at 23–27 weeks, LUS showed a low diagnostic accuracy with higher cut-off scores to predict msBPD on D3 (AUROC curve: 0.66, 95% CI: 0.51–0.79), D7 (AUROC curve: 0.69, 95% CI: 0.54–0.83), D14 (AUROC curve: 0.70, 95% CI: 0.54–0.83), and D21 (AUROC curve: 0.70, 95% CI: 0.54–0.83) (Table 2). Higher LUSs were observed in infants who developed msBPD from 3 days onwards, without significant differences compared to those in infants with no/mild BPD (Fig. 2E). The LUS evolution which predicted using the mixed-effects regression model is shown in Fig. 2F, and it showed the presence of persistently high LUS within 3 weeks after birth, regardless of their subsequent evolution to msBPD.

In the five-factor model, lower GA was consistently associated with the severity of BPD regardless of the postnatal day (D3, OR: 0.19, 95% CI: 0.04–0.85, *P* < 0.05; D7, OR: 0.14, 95% CI: 0.03–0.68, *P* < 0.05) (Table 3). The AUROC curve for GA to predict msBPD was 0.75 (95% CI: 0.59–0.85), and it provided diagnostic accuracy similar to that of LUSs, GA-adjusted LUSs, and the clinical five-factor model for all three time points (Delong’s test, overall *P* > 0.05; Table 4).

## Discussion

This study found that an early LUS, in the first 2 weeks of postnatal age, can predict any BPD and msBPD in infants with GA < 32 weeks. The study also showed that the contribution of LUS differed between extremely preterm infants and preterm infants born at 28–32 weeks of gestation. LUSs provided similar moderate predictive performance as GA-adjusted LUS and clinical multivariate models in infants born after 28 weeks, while LUSs seem to be less helpful in infants born before 28 weeks.

We followed a protocol and BPD definition similar to that of Loi et al. [9] and Alonso-Ojembarrena and Lubián-López [18]. Ojembarrena and Lubián-López [18] and Alonso-Ojembarrena et al. [20] reported that an LUS of ≥ 5 on D14 was the best predictor of any form of BPD (sensitivity: 74%, specificity: 100%, AUROC curve: 0.93, 95% CI: 0.80–0.99), and the optimal time for msBPD prediction was at 1 week of age, with a cut-off point of 8 (sensitivity: 70%, specificity: 79%, AUROC curve: 0.79, 95% CI: 0.74–0.84). In contrast, the present study had a similar AUROC curve to that of Alonso-Ojembarrena et al. [20] but a lower cut-off point. This difference may be due to either the intercenter variability in oxygen administration and the decision for respiratory support or the higher proportion of infants with msBPD in the total sample (42% vs 24.5%).

In infants born at 28–32 weeks of gestation, LUSs performed well in predicting the development of msBPD, similar to that observed in the total sample. LUSs decreased rapidly during the first week of life and remained high and stable in infants with evolving msBPD, as previously reported [12, 21]. Interestingly, the present study found that the predictive performance of the clinical five-factor model was superior to that of LUSs on D14. Thus, reducing the frequency of lung ultrasonography in these infants is possible if other studies verify this result. However, Alonso-Ojembarrena et al. [20] found that the diagnostic performance of their LUSs was comparable to that of the clinical multivariate model that included GA, sex, prenatal corticosteroids, surfactant, and invasive mechanical ventilation at D7, and late-onset sepsis before D7, and Loi et al. [9] showed that GA-adjusted LUS had slightly higher reliability than the NIH calculator. The authors thought that a possible explanation was the enrolment of relatively mature preterm infants (≥ 28 weeks) in current analysis. In addition, the authors believed that this difference may have been due in part to the choice of expanded LUS protocols that considered the posterior lung fields. BPD is a dis-homogeneous lung disease characterized by impaired alveolar structure and lung vascular growth [22]. Theoretically, with impaired ventilation in the gravity-dependent posterior lung areas, the probability of atelectasis is significantly increased, thereby increasing LUS. Therefore, a fuller representation of the lateral and posterior fields of the lung may be more accurate for the prediction of msBPD in very preterm infants. However, the additional value of adding posterior lung fields to the LUS in predicting BPD remains controversial. Loi et al. [9] and Liu et al. [8, 23] demonstrated that the predictive performance of expanded LUS protocols (10-region LUS and 12-region LUS) was superior to that of the 6-region protocol, and a meta-analysis and two prospective multicenter studies concluded that the diagnostic accuracies of LUS and expanded LUS for BPD and msBPD were similar [20, 24]. We believe it is valuable to question whether scanning of the posterior lung zones improves diagnostic accuracy, and national multicenter LUS data should be integrated to explore and optimize lung scanning protocols.

Alonso-Ojembarrena et al. [12] and Raimondi et al. [21] reported different LUS patterns from birth until 6–14 weeks in infants born before 28 weeks. Persistently high LUSs in the first 4 weeks after birth for infants with GA < 28 weeks were described, regardless of their subsequent evolution to msBPD. This may be related to the non-specificity of LUSs and the impact of frequent acute clinical events (inflammation and complications associated with respiratory support) due to respiratory insufficiency of prematurity. These results were consistent with those of our study. However, we observed no significant differences between infants born at 23–27 weeks of gestation with no/mild BPD and msBPD on D3 and D7 in our study. The effect of GA on LUSs and the high incidence of BPD in this group may have influenced the results. In infants born before 28 weeks, the predictive performance of LUSs was lower than that of infants with GA ≥ 28 weeks, and this difference was due to the persistently high LUSs in the more immature infants. GA remained the dominant predictor of msBPD, and adding LUSs or other clinical predictors in the first 2 weeks only marginally increased the AUROC curve; they were insufficient to reach statistical significance at any time point. Woods et al. [11] prospectively enrolled 100 infants born at < 28 weeks of gestation and reported that LUS on D3–D4 and D7 accurately predicted BPD (according to Jensen et al. [19]). However, lung ultrasonography performed within the first week of life has little value for GA-based models [11]. The AUROC curve for GA in predicting BPD in our cohort was 0.75; this was in alignment with their results, albeit with slightly higher AUROC curve values. Additionally, our results could suggest that the contribution of LUSs in this population with a higher BPD prevalence remains controversial. Mohamed et al. [10] and Abdelmawla et al. [25] reported that the AUROC curve for LUSs in the first 2 weeks after birth was as high as 0.95. The generalizability of these studies is limited owing to the impact of variability in respiratory management among different NICUs on the incidence of msBPD and the small sample size.

This study had certain limitations. First, although our data are in close agreement with that of other reports, this was only a single-center pragmatic study, which lacks the scientific rigor required to support widespread changes in practice. In addition, the intercenter variability in oxygen administration and decisions for respiratory support can significantly affect the generalizability of our results; thus, these findings need to be interpreted cautiously. Second, 6-h pronation has been reported to significantly improve lung aeration and gas exchange in infants with evolving BPD, resulting in decreased LUSs [26]. In our study, all infants were placed supine for the lung scanning, and the precise time of supine or prone position prior to lung ultrasonography was not recorded for each infant, which was likely to affect LUS results. Another limitation of our study is the small proportion of infants born before 28 weeks, and future studies should focus on the predictive value of LUS in this population.

## Conclusion

The collective results of available studies have shown that LUS has great promise for clinical research and the management of preterm infants. However, we must recognize that the contribution of LUS to predict msBPD in infants born before and after 28 weeks is uncertain. Hence, multicenter prospective studies that focus on the predictive performance of LUS data in infants of different GAs are necessary.

## Supplementary Information

Below is the link to the electronic supplementary material.

Supplementary file1 (DOCX 23 KB)

## Data Availability

All data generated or analyzed during this study are included in this article and its supplementary material files. Further enquiries can be directed to the corresponding author.

## References

[1] Onland W, Debray TP, Laughon MM, Miedema M, Cools F, Askie LM, Asselin JM, Calvert SA, Courtney SE, Dani C (2013). Clinical prediction models for bronchopulmonary dysplasia: a systematic review and external validation study. BMC Pediatr.

[2] El Faleh I, Faouzi M, Adams M, Gerull R, Chnayna J, Giannoni E, Roth-Kleiner M (2021) Bronchopulmonary dysplasia: a predictive scoring system for very low birth weight infants. A diagnostic accuracy study with prospective data collection. Eur J Pediatr 180(8):2453–2461. 10.1007/s00431-021-04045-8 10.1007/s00431-021-04045-8 PMC8285318 33822247

[3] Laughon MM, Langer JC, Bose CL, Smith PB, Ambalavanan N, Kennedy KA, Stoll BJ, Buchter S, Laptook AR, Ehrenkranz RA (2011). Prediction of bronchopulmonary dysplasia by postnatal age in extremely premature infants. Am J Respir Crit Care Med.

[4] Corsini I, Parri N, Ficial B, Dani C (2020). Lung ultrasound in the neonatal intensive care unit: review of the literature and future perspectives. Pediatr Pulmonol.

[5] Raimondi F, Yousef N, Rodriguez Fanjul J, De Luca D, Corsini I, Shankar-Aguilera S, Dani C, Di Guardo V, Lama S, Mosca F (2019). A multicenter lung ultrasound study on transient tachypnea of the neonate. Neonatology.

[6] Brat R, Yousef N, Klifa R, Reynaud S, Shankar Aguilera S, De Luca D (2015) Lung ultrasonography score to evaluate oxygenation and surfactant need in neonates treated with continuous positive airway pressure. JAMA Pediatr 169(8):e151797. 10.1001/jamapediatrics.2015.1797 10.1001/jamapediatrics.2015.1797 26237465

[7] Sharma D, Farahbakhsh N (2019). Role of chest ultrasound in neonatal lung disease: a review of current evidences. J Matern Fetal Neonatal Med.

[8] Liu X, Lv X, Jin D, Li H, Wu H (2021). Lung ultrasound predicts the development of bronchopulmonary dysplasia: a prospective observational diagnostic accuracy study. Eur J Pediatr.

[9] Loi B, Vigo G, Baraldi E, Raimondi F, Carnielli VP, Mosca F, De Luca D (2021) Lung ultrasound to monitor extremely preterm infants and predict bronchopulmonary dysplasia. A multicenter longitudinal cohort study. Am J Respir Crit Care Med 203(11):1398–1409. 10.1164/rccm.202008-3131OC 10.1164/rccm.202008-3131OC 33352083

[10] Mohamed A, Mohsen N, Diambomba Y, Lashin A, Louis D, Elsayed Y, Shah PS (2021). Lung ultrasound for prediction of bronchopulmonary dysplasia in extreme preterm neonates: a prospective diagnostic cohort study. J Pediatr.

[11] Woods PL, Stoecklin B, Woods A, Gill AW (2021). Early lung ultrasound affords little to the prediction of bronchopulmonary dysplasia. Arch Dis Child Fetal Neonatal Ed.

[12] Alonso-Ojembarrena A, Montero-Gato J, Gregorio-Hernández R, Aldecoa-Bilbao V, Alonso-Quintela P, Rodriguez-Fanjul J, Concheiro-Guisán A, Trujillo-Fagundo A, García-Ojanguren AM, de Las H-M (2022). Lung ultrasound scores progress differently in extreme and very preterm infants after birth: a multicentre prospective study. Neonatology.

[13] Kurepa D, Zaghloul N, Watkins L, Liu J (2018). Neonatal lung ultrasound exam guidelines. J Perinatol.

[14] Mirza H, Garcia J, McKinley G, Hubbard L, Sensing W, Schneider J, Oh W, Wadhawan R (2019). Duration of significant patent ductus arteriosus and bronchopulmonary dysplasia in extremely preterm infants. J Perinatol.

[15] Schena F, Francescato G, Cappelleri A, Picciolli I, Mayer A, Mosca F, Fumagalli M (2015). Association between hemodynamically significant patent ductus arteriosus and bronchopulmonary dysplasia. J Pediatr.

[16] Dani C, Lista G, Bianchi S, Mosca F, Schena F, Ramenghi L, Zecca E, Vento G, Poggi C, Leonardi V (2021). Intravenous paracetamol in comparison with ibuprofen for the treatment of patent ductus arteriosus in preterm infants: a randomized controlled trial. Eur J Pediatr.

[17] Jobe AH, Bancalari E (2001). Bronchopulmonary dysplasia. Am J Respir Crit Care Med.

[18] Alonso-Ojembarrena A, Lubián-López SP (2019). Lung ultrasound score as early predictor of bronchopulmonary dysplasia in very low birth weight infants. Pediatr Pulmonol.

[19] Jensen EA, Dysart K, Gantz MG, McDonald S, Bamat NA, Keszler M, Kirpalani H, Laughon MM, Poindexter BB, Duncan AF et al (2019) The diagnosis of bronchopulmonary dysplasia in very preterm infants. An evidence-based approach. Am J Respir Crit Care Med 200(6):751–759. 10.1164/rccm.201812-2348OC 10.1164/rccm.201812-2348OC PMC6775872 30995069

[20] Alonso-Ojembarrena A, Serna-Guerediaga I, Aldecoa-Bilbao V, Gregorio-Hernández R, Alonso-Quintela P, Concheiro-Guisán A, Ramos-Rodríguez A, de Las H-M, Rodeño-Fernández L, Oulego-Erroz I (2021). The predictive value of lung ultrasound scores in developing bronchopulmonary dysplasia: a prospective multicenter diagnostic accuracy study. Chest.

[21] Raimondi F, Migliaro F, Corsini I, Meneghin F, Dolce P, Pierri L, Perri A, Aversa S, Nobile S, Lama S et al (2021) Lung ultrasound score progress in neonatal respiratory distress syndrome. Pediatrics 147(4). 10.1542/peds.2020-030528 10.1542/peds.2020-030528 33688032

[22] Loi B, Casiraghi C, Catozzi C, Storti M, Lucattelli M, Bartalesi B, Yousef N, Salomone F, De Luca D (2021) Lung ultrasound features and relationships with respiratory mechanics of evolving BPD in preterm rabbits and human neonates. J Appl Physiol (Bethesda, Md: 1985) 131(3):895–904. 10.1152/japplphysiol.00300.2021 10.1152/japplphysiol.00300.2021 34292788

[23] Liu J, Copetti R, Sorantin E, Lovrenski J, Rodriguez-Fanjul J, Kurepa D, Feng X, Cattaross L, Zhang H, Hwang M et al (2019) Protocol and guidelines for point-of-care lung ultrasound in diagnosing neonatal pulmonary diseases based on international expert consensus. J Vis Exp JoVE (145). 10.3791/58990 10.3791/58990 30907892

[24] Pezza L, Alonso-Ojembarrena A, Elsayed Y, Yousef N, Vedovelli L, Raimondi F, De Luca D (2022). Meta-analysis of lung ultrasound scores for early prediction of bronchopulmonary dysplasia. Ann Am Thorac Soc.

[25] Abdelmawla M, Louis D, Narvey M, Elsayed Y (2019). A lung ultrasound severity score predicts chronic lung disease in preterm infants. Am J Perinatol.

[26] Loi B, Regiroli G, Foligno S, Centorrino R, Yousef N, Vedovelli L, De Luca D (2022) Respiratory and haemodynamic effects of 6h-pronation in neonates recovering from respiratory distress syndrome, or affected by acute respiratory distress syndrome or evolving bronchopulmonary dysplasia: a prospective, physiological, crossover, controlled cohort study. eClinicalMedicine 101791. 10.1016/j.eclinm.2022.101791 10.1016/j.eclinm.2022.101791 PMC9874350 36712892

