# Supplementary material for: Comparison of lung ultrasound scores with clinical models for predicting bronchopulmonary dysplasia
Source: Eur J Pediatr. 2023 Feb 9;182(4):1697–705. doi: 10.1007/s00431-023-04847-y (PMC10167145; doi:10.1007/s00431-023-04847-y)
Supplement: Supplementary file 1 — Supplementary file1 (DOCX 23 KB) [file 431_2023_4847_MOESM1_ESM.docx]

Supplement Table 1. clinical characteristics of the included patients according to GA at birth

| Characteristic | 24-27 weeks  (n=43) | 28-32 weeks  (n=107) |
| --- | --- | --- |
| GA, wk, median (IQR) | 26.6(26.0-27.4) | 29.6(28.9-30.3) |
| BW median (IQR), g | 960(840-1050) | 1280(1120-1425) |
| Male, No. (%) | 23(53.5) | 54 (50.5) |
| SGA neonates, No. (%) | 0(0) | 5(4.5) |
| Cesarean delivery, No. (%) | 4(9.3) | 53(49.5) |
| CRIB-II Score, median (IQR) | 11(10-12) | 7 (5-8) |
| Antenatal steroids, No. (%) | 27(62.8) | 70 (65.4) |
| PPROM, No. (%) | 14(32.6) | 39(36.4) |
| MV for over 24h, No. (%) | 41(95.3) | 66(61.7) |
| Clinical chorioamnionitis, No. (%) | 15 (34.9) | 19 (17.8) |
| hsPDA | 41(95.3) | 49(44.5) |
| msBPD | 33(76.7) | 30(27.2) |

Definition of abbreviations: msBPD=moderate to severe bronchopulmonary dysplasia; hsPDA= hemodynamically significant patent ductus arteriosus; IQR=interquartile range; MV= Mechanical ventilation; PPROM=preterm premature rupture of membranes; SGA=small for gestational age.

Supplement Table 2. The predictive ability of LUS for BPD in infants born at 23-32 weeks

|  | Cut off  point | AUC (95%CI) | Sensitivity (95%CI) | Specificity (95%CI) | +LR (95%CI) | -LR (95%CI) | PPV (95%CI) | NPV (95%CI) |
| --- | --- | --- | --- | --- | --- | --- | --- | --- |
| D3 | ＞4 | 0.77(0.69-0.83) | 74.8% (65.4 - 82.7) | 74.4% (58.8 - 86.5) | 2.92 (1.7 - 4.9) | 0.34 (0.2 - 0.5) | 74.5% (63.4 - 83.1) | 74.7% (67.1 - 81.0) |
| D7 | ＞4 | 0.78(0.70-0.84) | 65.4% (55.6 - 74.4) | 76.7% (61.4 - 88.2) | 2.81 (1.6 - 4.9) | 0.45 (0.3 - 0.6) | 73.8% (61.6 - 83.1) | 68.9% (62.0 - 75.1) |
| D14 | ＞4 | 0.87 (0.81-0.92) | 72.9% (63.4 - 81.0) | 90.70% (77.9 - 97.4) | 7.84 (3.1 - 20.1) | 0.30 (0.2 - 0.4) | 88.7% (75.4 - 95.3) | 77.0% (70.7 - 82.2) |
| D21 | ＞5 | 0.81(0.74-0.87) | 76.2% (63.8 - 86.0) | 72.4% (61.8 - 81.5) | 2.76(1.9 - 4.0) | 0.33(0.2-0.5) | 66.7% (58.1- 4.3) | 80.8% (72.6-86.9) |

Definition of abbreviations: AUC＝area under the receiver operating characteristic curve; CI=confidence interval; LR=likelihood ratio; PPV=positive predictive values; NPV=negative predictive values.

Supplement Table 3. Statistics of the linear multilevel mixed-effects regression models selected for LUS evolution. b1-2 create a knot point at seven days of life.

|  | **Coefficient** | **Standard error** | **z** | **p** |
| --- | --- | --- | --- | --- |
| **Model 1: 23-27 weeks** | |  |  |  |
| Constant | 7.14 | 0.79 | 9.08 | <0.001 |
| a1 | -0.005 | 0.06 | -0.09 | 0.93 |
| msBPD | 1.20 | 0.90 | 1.34 | 0.18 |
| a1x msBPD | 0.05 | 0.07 | 0.75 | 0.45 |
| **Model 4: 28-32 weeks** | |  |  |  |
| Constant | 5.87 | 0.41 | 14.47 | <0.001 |
| b1 | -0.28 | 0.08 | -3.70 | <0.001 |
| b2 | -0.05 | 0.03 | -2.12 | 0.035 |
| msBPD | 3.80 | 1.18 | 2.23 | <0.001 |
| b1xmsBPD | -0.20 | 0.14 | -1.38 | 0.17 |
| b2x msBPD | 0.09 | 0.05 | 1.89 | 0.06 |

Supplement Table 4. multicollinearity diagnosis of clinical covariates

| **28-32 weeks** | D3 | | | | | |  | | | | D7 | | | | | | | |  | | | | | D14 | | |
| --- | --- | --- | --- | --- | --- | --- | --- | --- | --- | --- | --- | --- | --- | --- | --- | --- | --- | --- | --- | --- | --- | --- | --- | --- | --- | --- |
|  | Variable | Tolerance | | VIF | |  | | Variable | | | | Tolerance | | | VIF |  | | | | | | Variable | | | Tolerance | VIF |
|  | GA | | 0.990 | 1.010 | |  | | GA | | | | 0.912 | | | 1.096 |  | | | | | | GA | | | 0.950 | 1.052 |
|  | Respiratory support | | 0.991 | 1.009 | |  | | Respiratory support | | | | 0.912 | | | 1.096 |  | | | | | | Respiratory support | | | 0.989 | 1.011 |
|  | PtcO2 | | 0.998 | 1.002 | |  | | PtcO2 | | | | 1.000 | | | 1.000 |  | | | | | | PtcO2 | | | 0.948 | 1.055 |
| **23-27 weeks** | D3 | | | | | |  | | | | D7 | | | | | | | |  | | | | | D14 | | |
|  | Variable | | Tolerance | | VIF |  | | | | Variable | | | Tolerance | | | | VIF | | |  | | | Variable | | Tolerance | VIF |
|  | GA | | 0.869 | 1.151 | | | | |  | | GA | | | 0.896 | | | | 1.116 | | |  | | GA | | 0.805 | 1.242 |
|  | Respiratory support | | 0.864 | 1.157 | | | | |  | | Respiratory support | | | 0.884 | | | | 1.132 | | |  | | Respiratory support | | 0.720 | 1.390 |
|  | PtcO2 | | 0.920 | 1.087 | | | | |  | | PtcO2 | | | 0.914 | | | | 1.094 | | |  | | PtcO2 | | 0.858 | 1.166 |

Definition of abbreviations: GA= gestational age; VIF= variance inflation factor.
